# Survey of preferred guideline attributes: what helps to make guidelines more useful for emergency health practitioners?

**DOI:** 10.1186/1865-1380-5-42

**Published:** 2012-11-10

**Authors:** Samar Aboulsoud, Sue Huckson, Peter Wyer, Eddy Lang

**Affiliations:** 1 Weill Cornell Medical College, Doha, Qatar; 2 Hamad Medical Corporation, P.O. Box 24250, Doha, Qatar; 3 Cairo University, Cairo, Egypt; 4 Australian and New Zealand Intensive Care Society (ANZICS), 10 Ievers Terrace, Carlton, Victoria, 3053, Australia; 5 Columbia University College of Physicians & Surgeons, 630 West 168th Street, New York, NY, USA; 6 Alberta Health Services, University of Calgary, Calgary, Alberta, Canada

## Abstract

**Background:**

Enhancing CPG acceptance and implementation can play a major role in the development and establishment of emergency medicine as a specialty in many parts of the world. A Guideline International Network special interest group established to support collaboration to improve uptake of clinical practice guidelines (CPGs) across the emergency care sector conducted an international survey to identify attributes of guideline likely to enhance their use.

**Methods:**

A Web-based survey was undertaken to determine how CPGs were accessed, the preferred formats and attributes of guidelines, and familiarity with GRADE. The criteria used to identify preferred attributes of guidelines were adapted from the AGREE II Tool.

**Results:**

Two hundred six responses were received from 31 countries, 74/206 (36%) from the US, 28/206 (16%) from Canada, 17/206 (8%) from Australia and 15/206 (7%) from the UK. The majority of responses were from physicians (176/206, 85%) with 15/206 (7%) of responses from nurses and 9/206 (4%) from pre-hospital emergency services personnel. The preferred format for guidelines was clinical protocols that incorporated recommendations into workflow, and the most preferred attribute of guidelines was the clear identification of key recommendations. The results also identified that within the group that responded to the question related to GRADE, 66% were unfamiliar with this system for summarizing evidence in relationship to recommendations.

**Conclusions:**

The findings provide the basis for further research to explore the most appropriate formats for guidelines or guidelines resources tailored to the needs of the emergency care providers.

## Background

In 2007 the Guidelines International Network (G-I-N) established the Emergency Care (EC) Community as a special interest group of G-I-N to support collaboration to improve both the awareness and uptake of clinical practice guidelines across the emergency care sector internationally. One of the first initiatives of this group was to seek to identify the attributes of guidelines and guideline resources to improve the usability and implementation of guideline recommendations.

The 2006 IOM report “Future of Emergency Care Series, Hospital Emergency Care at the Breaking Point” called for the standardization and implementation of guidelines [1]. This report was based on the a comprehensive independent review that focused on all aspects of emergency care provided by US Emergency Medical Services. The findings of the review have had wide applicability to emergency services internationally. The report identified the increasing importance of guidelines and their implementation to improve patient care and health outcomes based on the best available research.

The gap in general awareness and implementation of guidelines has been well documented over the last 2 decades with specific reference to emergency medicine by Schriger et al. in 1993 [2-5]. Enhancing guideline applicability acceptance and implementation can play a major role in the development of emergency medicine as a specialty in many parts of the world. The EC Community sought to conduct a scoping survey as a hypothesis-generating effort to determine aspects of attitudes, preferences and awareness of guidelines across various professional groups of Emergency Medical Service (EMS) providers.

## Methods

A Web-based survey (Attachment A) was undertaken from 14 November 2010 to 15 December 2010. The survey was developed by a working group of the G-I-N EC Community with questions focusing on:

1) how CPGs are accessed,

2) which guideline formats and attributes are preferred, and

3) the degree of respondent familiarity with GRADE (Grading of Recommendations Assessment, Development and Evaluation).

The criteria used to identify preferred attributes of guidelines were adapted from the AGREE II Tool (Appraisal of Guidelines Research and Evaluation Tool) [6]. The working group also considered it important to gauge the knowledge related to the GRADE system. GRADE is being widely adopted to inform the strength of recommendations based on a transparent explicit approach for rating quality of evidence and connecting evidence to recommendations [7]. The International Liaison Committee on Resuscitation (http://www.ilcor.org), the premier organization for developing evidence-based guidance on emergency care, is transitioning to GRADE in 2015.

The survey (Appendix A) was piloted widely through the EC Community membership. Further feedback was also sought from G-I-N board members. Changes made to the survey included revising the Likert rating to remove a neutral response and ensure that all professional groups were considered in the demographic information. The Australasian College for Emergency Medicine framework was used to classify practice settings based on the Emergency Departments’ level of service available [8].

A snowballing technique was used to distribute the survey; this method relies on referrals from initially sampled respondents to others who are believed to have the same interest [9]. The survey was e-mailed by the working group and the EC Community members worldwide to utilize their extended networks across medical, nursing and pre-hospital professional groups to invite further participation. The survey was limited to English-speaking emergency healthcare providers from primary and tertiary care including urban and rural settings.

Frequency tables were used to determine the results of from the survey questions. A SPSS package was used to analyze the data. The chi-square test was used to analyze several associations: (1) length of practice and use of guidelines in daily practice, (2) practice setting and preferred guideline formats and (3) practice setting and methods of accessing of guidelines.

Aggregation of the practice setting into community level care and tertiary level care that included the major referral and major regional emergency departments was done to increase the sample size. The responses were aggregated to provide more meaningful interpretation (agree and strongly agree were considered as one category, as was disagree and strongly disagree) [10]. Associations between GRADE familiarity and its usefulness were also analyzed.

## Results

### Demographics

Two hundred six responses were received from 31 countries with representation from the US, UK, Canada and Australia. The majority of responses were from physicians (176/206, 85%) with 15/206 (7%) of responses from nurses and 9/206 (4%) from pre-hospital emergency services personnel.

Practice experience of responding emergency practitioners ranged from less than 2 years to greater than 20 years. Of the respondents, 144/206 (16%) were from tertiary-level health facilities, 14/206 (16%) from community- or urban-based healthcare facilities settings and 30/206 (15%) from rural or maritime settings.

### Accessing guidelines

**Figure 1:**
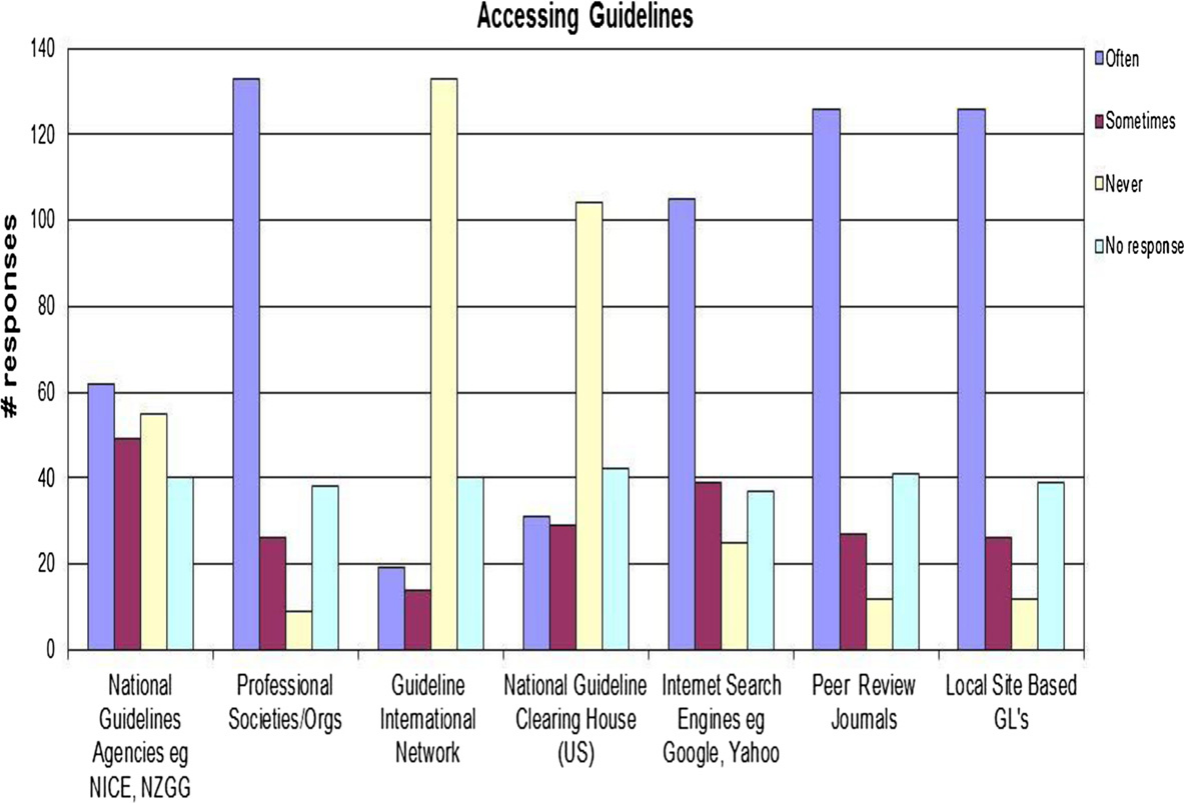
Responses to where guidelines are sourced to inform practice.

The responses identified that the most common sources for guidelines are from professional societies and peer review journals as shown in Figure 1.

**Figure 2:**
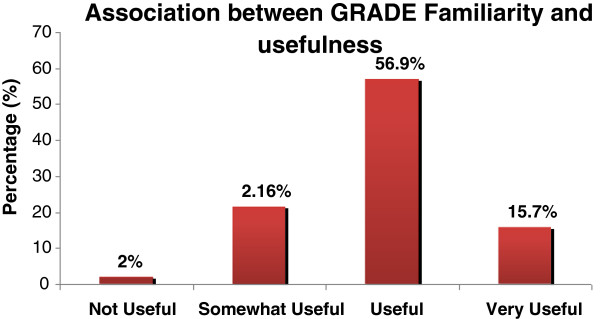
Association between GRADE familiarity and usefulness.

Table 1 summarizes the survey results related to length of respondent experience in the emergency care sector, their use of guidelines in daily practice, preferred formats of guidelines and their familiarity with GRADE. Of those who endorsed familiarity with GRADE, 56.9% reported that they found it useful and 15.7% found it as very useful. Figure 2 illustrates the associations between GRADE familiarity and its usefulness. Table 2 summarizes the preferred attributes of guidelines as adapted from the AGREE II Tool. All responses were rated 97% or above.

**Table 1:** Summary of survey responses

**Length of practice in the emergency care sector**	**% Responses**
Up to 2 years	(20/170) **10%**
From 2 to 5 years	(27/170) **13%**
From 5 to 10 years	(45/170) **22%**
From 10 to 15 years	(36/170) **17%**
From 15 to 20 years	(39/170) **19%**
Greater than 20 years	(39/170) **19%**
**Is the use of clinical practice guidelines a part of your daily practice?**	**% Responses**
Always use guidelines	(37/170) **22%**
Usually use guidelines	(81/170) **48%**
Occasionally use guidelines	(50/170) **29%**
Guidelines not used or discussed	(2/170) **1%**
**Preferred formats of guidelines or guideline resources to support uptake of best practice at point of care?**	**% Agree responses**
Clinical protocols that translate recommendations into work flow	(150/168) **85%**
Plain language evidence summaries	(118/164) **72%**
Clinical algorithms (flow charts) formats	(120/167) **72%**
Electronic order sets with incorporating guidelines	(109/164) **65%**
Prioritized list of recommendations, e.g., Care Bundles	(95/164) **58%**
Full systematic reviews	(94/167) **56%**
Education slide sets	(76/167) **46%**
**Grading the evidence**	**% Agree responses**
Are you familiar with GRADE?	(99/151) **66%**

**Table 2:** The preferred attributes of guidelines as adapted from the AGREE II Tool

**Preferred attributes of guidelines/guideline resources**	**% Agree responses**
1. The overall objective(s) of the guideline is (are) specifically described	(146/146) **100%**
2. The health question(s) covered by the guideline is (are) specifically described	(144/146) **99%**
3. The population (patients, public, etc.) to whom the guideline is meant to apply is specifically described	(146/146) **100%**
4. The guideline development group includes individuals from all the relevant professional groups	(146/146) **100%**
5. The views and preferences of the target population (patients, public, etc.) have been sought	(146/146) **100%**
6. The target users of the guideline are clearly defined	(144/146) **99%**
7. Systematic methods were used to search for evidence	(143/145) **99%**
8. The criteria for selecting the evidence are clearly described	(143/144) **99%**
9. The strengths and limitations of the body of evidence are clearly described	(143/143) **100%**
10. The methods for formulating the recommendations are clearly described	(141/141) **100%**
11. The health benefits, side effects and risks have been considered in formulating the recommendations	(143/145) **99%**
12. There is an explicit link between the recommendations and the supporting evidence	(143/145) **99%**
13. The guideline has been externally reviewed by experts prior to its publication	(143/146) **98%**
14. A procedure for updating the guideline is provided	(142/145) **98%**
15. The recommendations are specific and unambiguous	(143/144) **99%**
16. The different options for management of the condition or health issue are clearly presented	(140/143) **98%**
17. Key recommendations are easily identifiable	(145/145) **100%**
18. The guideline describes facilitators and barriers to its application	(143/143) **100%**
19. The guideline provides advice and/or tools on how the recommendations can be put into practice	(141/145) **97%**
20. The potential resource implications of applying the recommendations have been considered	(143/143) **100%**
21. The guideline presents (includes) monitoring and/ or auditing criteria	(145/145) **100%**
22. The views of the funding body have not influenced the content of the guideline	(143/145) **99%**
23. Competing interests of guideline development group members have been recorded and addressed	(140/144) **97%**

## Discussion

This study has provided data from the emergency care practice environment related to the behaviors, attitudes, preferences and knowledge about CPGs. Barriers to the uptake of CPGs across these domains were first reported by Cabana in 1999 [3]; many of those issues are still prevalent today in a context of many more guidelines being published on a daily basis.

The results of this study support previous research that identified that guideline formats and content are important determinants of usability of guidelines in busy clinical environments such as the emergency care sector [11-13]. A survey of multidisciplinary clinicians in Australian public hospitals reported that concise, quick-reference formats were preferred to detailed texts (35% vs. 6%; *P* < 0.001) [12-14]. The preferred format for guidelines identified through this survey was clinical protocols that incorporated recommendations into workflow. Understanding the practice environment is a critical aspect to consider when seeking to enhance the usability of guidelines.

The most preferred guideline attribute identified through this survey was the clear identification of key recommendations. The preferences support information related to ‘what to do, why and to whom.’ Knowledge of the CPG development process was also listed as important; the systematic approach taken to review the evidence provides a level of confidence and authority in the recommendations made.

Professional societies and peer review journals were the most frequently used sources to access CPGs. G-I-N and the National Guideline Clearinghouse (NGC) are large web-based repositories of guidelines, which were almost never used to source guidelines among the respondents; some of the reasons for this could be a lack of knowledge of these repositories or that health providers have a higher level of assurance of the quality of guidelines that are published in the peer review literature.

There was no evidence of an association between the level of service provided, e.g., tertiary care or community-based care, preferred formats or where the CPGs were accessed. However, there was a positive association between more years of practice within the setting and use of CPGs. The value of guidelines as a means of establishing standards of care and reducing variation in practice may be better appreciated as more experience is gained working within the health systems.

As the guideline development process continues to evolve, there has been an emergence of grading systems such as GRADE, which is gaining some prominence. This study revealed that 66% of the respondents were unfamiliar with the GRADE system for rating the quality of evidence and recommendations. This is supported by Kotzeva et al. [13-15], who reported that clinicians had limited knowledge, experience and understanding of GRADE. This knowledge deficit related to the grading of recommendations is potentially important as a reflection of respondent capacity to recognize and critically evaluate the approach to rating the strength of evidence used by recently developed guidelines.

## Conclusions

This study is the first to explore the preferred attributes of guidelines of those emergency care practitioners who work in an environment that is unique in the diversity of presentations often requiring time-critical decisions on management needing access to relevant high-quality information. The findings suggest the need for further research to explore the most appropriate formats for guidelines or guideline resources tailored to both the needs of the specific setting and healthcare providers within that setting. They also support the usefulness of educational efforts to increase awareness and literacy in emergency systems of grading evidence and recommendations such as GRADE.

**Figure 3:**
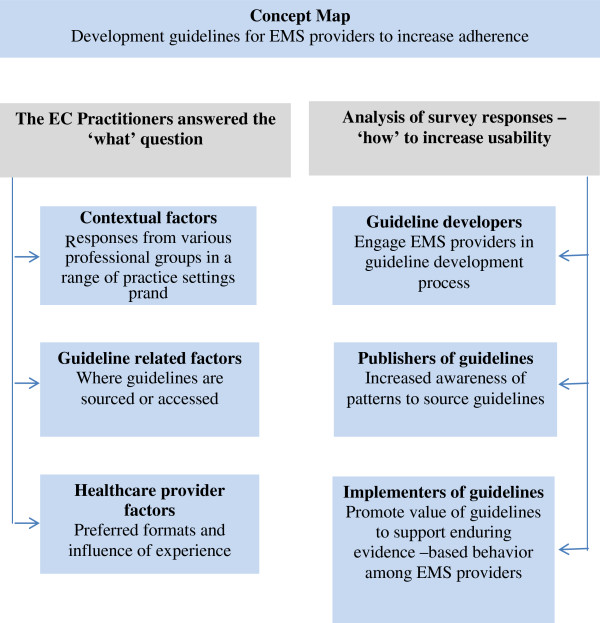
Concept map to illustrate strategies to increase emergency medical services (EMS) providers’ adherence to guidelines in response to the themes arising from the survey.

Although the results of this survey largely reflect the attitudes of health professionals in well-resourced countries, the following concepts, illustrated in Figure 3, have potentially broad applicability across emergency care services:

Guideline developers need to engage with end-users to ensure that guideline formats and information are relevant for specific settings and tailored to needs of EMS providers

Publishers of guidelines need to be aware of EMS provider behaviors for accessing guidelines, and

Promotion of guidelines to EMS providers at point of care is central to their practice to support enduring evidence-based behaviors.

### Limitations

There are a number of limitations of this study. The distribution of respondents was very heavily biased toward physicians practicing in a tertiary care setting. The sample was predominantly from the US, UK, Australia and Canada with minimal representation from developing countries. The process used was nonrandom selection of participants and relied on the subjective judgments of respondents.

### Appendix A

#### G-I-N Emergency Care Survey 2010

##### Demographics

We would like to know a little information about you.

Please select your professional status from the following list:

❒ Physician

❒ Physician assistant

❒ Nurse

❒ Nurse assistant

❒ Emergency services personnel (pre-hospital)

❒ Other

If other, please specify:

Please select which timeframe best describes the period you have been working in or have worked in the emergency care sector.

❒ Up to 2 years

❒ From 2 to 5 years

❒ From 5 to 10 years

❒ From 10 to 15 years

❒ From 15 to 20 years

❒ Greater than 20 years

Please state in which country you predominantly practice.

Please select which best describes the practice environment that you predominantly work in:

❒ Major referral emergency department (tertiary care)

❒ Urban district emergency department (community care)

❒ Major regional/rural base emergency department (tertiary care)

❒ Rural emergency service (community care)

❒ Primary care/remote rural emergency service

❒ Other

If other, please describe.

#### Preferred formats of guidelines

The following questions relate to your preferred formats of guidelines or other documents that incorporate evidence-based recommendations drawn from guidelines that have been developed from the best available evidence/research.

Is the use of clinical practice guidelines a part of your daily practice?

What are your preferred formats of guidelines or guideline resources to support uptake of best practice at point of care?

- Full systematic review with evidence tables and associated recommendations

- Clinical protocols that translate evidence-based recommendations into a desired workflow or process.

- Plain language ‘Evidence into Practice’ resources that summarize the evidence and implications for practice.

- Clinical algorithms or flow charts that provide a step-by-step decision-support tool

- Educational materials to promote the use of evidence-based recommendations in practice

Please select the answer that best describes where you access guidelines to inform your practice:

- Government-based agencies, e.g., NICS, NHMRC, SIGN, New Zealand Guideline Group, Singapore Ministry of Health, etc.

- From professional specialties or specialty organizations, e.g., the American College of Emergency Physicians (ACEP), International Resuscitation Council, British Thoracic Society, etc.

- From the Guideline International Network (G-I-N) Library

- From the National Guideline Clearing House (NGC)

- Use of the Internet search function, e.g., Google, Yahoo, etc.

- Peer review journals

- Locally developed guidelines

- Other (please specify)

#### Preferred attributes of guidelines/guideline resources

For the following questions, please consider what information within a guideline is important for you at point of care. These questions are adapted from the criteria used by AGREE II (Appraisal of Guidelines Research & Evaluation). AGREE II is an internationally validated tool for the assessment of clinical practice guidelines; http://www.agreetrust.org/.

Scope and purpose

- The overall objective(s) of the guideline is (are) specifically described.

- The health question(s) covered by the guideline is (are) specifically described.

- The population (patients, public, etc.) to whom the guideline is meant to apply is specifically described.

Stakeholder involvement

The guideline development group includes individuals from all the relevant professional groups.

- The views and preferences of the target population (patients, public, etc.) have been sought.

- The target users of the guideline are clearly defined.

Rigor of development

- Systematic methods were used to search for evidence.

- The criteria for selecting the evidence are clearly described.

- The strengths and limitations of the body of evidence are clearly described.

- The methods for formulating the recommendations are clearly described.

- The health benefits, side effects and risks have been considered in formulating the recommendations.

- There is an explicit link between the recommendations and the supporting evidence.

- The guideline has been externally reviewed by experts prior to its publication.

- A procedure for updating the guideline is provided.

Clarity and presentation

- The recommendations are specific and unambiguous

- The different options for management of the condition or health issue are clearly presented.

- Key recommendations are easily identifiable.

Applicability (implementability of the guideline or guideline product)

- The guideline describes facilitators and barriers to its application.

- The guideline provides advice and/or tools on how the recommendations can be put into practice.

The potential resource implications of applying the recommendations have been considered.

The guideline presents (includes) monitoring and/or auditing criteria.

Editorial independence.

The views of the funding body have not influenced the content of the guideline.

Competing interests of guideline development group members have been recorded and addressed.

#### Grading the evidence

Are you familiar with the GRADE system to assess guideline recommendations?

❒ Yes

❒ No

If your answer is yes to the above question, how useful do you find the GRADE assessment

❒ Very useful

❒ Useful

❒ Neutral

❒ Useful sometimes

❒ Not useful at all

### Competing interest

The authors declare that they have no competing interests.

### Authors’ contributions

SA is lead author and provided statistical analysis. SH was co-author and reviewed the manuscript. PW reviewed and contributed editorial advice to the article. EL reviewed and contributed editorial advice. All authors read and approved the final manuscript.

### Disclaimer

This article reports on the work of the EC Community and does not reflect the views of G-I-N. The EC Community supported G-I-N through the provision of secretarial support and a small grant. The Guidelines International Network (G-I-N; http://www.g-i-n.net) is a Scottish Charity recognized under Scottish Charity No. SC034047.
